# Feasibility and Effects of Digital Interventions to Support People in Recovery From Substance Use Disorders: Systematic Review

**DOI:** 10.2196/jmir.9873

**Published:** 2018-08-23

**Authors:** Sverre Nesvåg, James R McKay

**Affiliations:** ^1^ Centre for Alcohol and Drug Research Stavanger University Hospital Stavanger Norway; ^2^ Department of Psychiatry Perelman School of Medicine University of Pennsylvania Philadelphia, PA United States; ^3^ Philadelphia VA Medical Center Philadelphia, PA United States

**Keywords:** digital interventions, substance use disorders, recovery support, feasibility, effects

## Abstract

**Background:**

The development and evaluation of digital interventions aimed at preventing or treating substance use–related problems and disorders is a rapidly growing field. Previous reviews of such interventions reveal a large and complex picture with regard to targeted users, use, and efficacy.

**Objective:**

The objective of this review was to investigate the feasibility and effects of interventions developed specifically for digital platforms. These interventions are focused on supporting people in recovery from substance use disorders by helping them achieve their substance use goals and develop a more satisfying life situation.

**Methods:**

The review is based on a systematic search in MEDLINE, Embase, PsycInfo, and Cochrane Library databases. Of the 1149 identified articles, 722 were excluded as obviously not relevant. Of the remaining articles, 21 were found to be previous reviews, 269 were on interventions aimed at reducing hazardous alcohol or cannabis use, and 94 were on digitized versions of standard treatment methods. The remaining 43 articles were all read in full and systematically scored by both authors.

**Results:**

The 43 articles cover 28 unique interventions, of which 33 have been published after 2013. The interventions are aimed at different target groups (defined by age, substance, or comorbidity). Based on the number of features or modules, the interventions can be categorized as simple or complex. Fourteen of the 18 simple interventions and 9 of the 10 complex interventions have been studied with quantitative controlled methodologies. Thirteen of the 18 simple interventions are integrated in other treatment or support systems, mainly delivered as mobile phone apps, while 6 of the 10 complex interventions are designed as stand-alone interventions, most often delivered on a platform combining desktop/Web and mobile phone technologies. The interventions were generally easy to implement, but in most cases the implementation of the complex interventions was found to be dependent on sustained organizational support. Between 70% and 90% of the participants found the interventions to be useful and easy to use. The rates of sustained use were also generally high, except for simple interventions with an open internet-based recruitment and some information and education modules of the complex interventions. Across all interventions, slightly more than half (55%) of the studies with control groups generated positive findings on 1 or more substance use outcomes, with 57% of the interventions also found to be efficacious in 1 or more studies. In the positive studies, effects were typically in the small to moderate range, with a few studies yielding larger effects. Largely due to the inclusion of stronger control conditions, studies of simple interventions were less likely to produce positive effects.

**Conclusions:**

The digital interventions included in this review are in general feasible but are not consistently effective in helping people in recovery from substance use disorder reduce their substance use or achieving other recovery goals.

## Introduction

Treatment for substance use disorders (SUD) can be effective. However, individuals who enter treatment often struggle with factors that are slow to change or do not change at all, placing them at heightened risk for relapse for considerable lengths of time [1]. These include genetic factors, interpersonal problems, co-occurring psychiatric disorders, employment problems, and various neurocognitive conditions [2-6]. Moreover, most positive factors associated with recovery, such as the development of supportive social networks, interests and passions that reinforce abstinence, improved coping responses, employment, and other activities that provide a sense of worth and self-esteem, are slow to change and require ongoing support to prevent deterioration [7-9].

These findings may explain why treatments derived from an acute care model are of limited effectiveness in the long-term management of SUD. Specifically, vulnerability to relapse remains relatively high for significant periods after standard treatment protocols of 3 to 6 months have ended [10,11]. Better management requires longer periods of continued contact with the patient [9,12-14] to address flagging motivation, increased craving, diminished participation in self/mutual help, limitations in neurocognitive function, continued biological vulnerability to stress, and various other problems that arise. Therefore, extended treatment, otherwise known as continuing care, is often recommended to patients.

In addition to continuing care interventions focused on substance use, there are additional sources of long-term recovery support including mutual help programs such as Alcoholics Anonymous. Individuals who attend these programs often have good substance use outcomes, but only a minority of people who might benefit actually attend any meetings and very few continue to participate at a high level over long periods of time [15,16].

There is some evidence that interventions to improve housing and employment status produce improved substance use outcomes. For example, work by Silverman and colleagues [17] has shown that a therapeutic workplace intervention improves substance use outcomes and employment status over periods as long as 5 years for homeless individuals with SUD. Milby and colleagues [18] found that adding abstinence-contingent housing and work therapy to standard care improved short-term substance use and housing outcomes for homeless cocaine-dependent individuals but not 12-month outcomes [19]. In a second study, Milby et al [20] found that housing, whether contingent on abstinence or not, produced better substance use outcomes than no housing out to 6 months. However, providing housing did not improve housing or employment outcomes over 12 months relative to the no-housing condition.

Evidence from well-done randomized studies also supports the efficacy of recovery check-ups and case management in the longer term management of SUD. Brief quarterly check-ups designed to identify individuals with out-of-control drug use following treatment and quickly re-engage them in SUD care have improved substance use outcomes over 4 years relative to standard care, although the magnitude of the effects was small [21]. Intensive case management provided over 12 months has been shown to improve substance use outcomes and employment in welfare recipients [22,23].

Although extended treatment for SUD is effective [24], the magnitude of the effects is often not large and tends to decrease over time [9]. There are several reasons for this:

- Information on relapse risk is only obtained during treatment sessions. Some relapse vulnerability factors can change rapidly—over periods as short as a few hours—often with little or no warning. A continuing care intervention in which data on relapse risks are obtained only during treatment sessions cannot be responsive to sudden shifts in risk level between sessions.
- Counselor availability is limited. Patients are urged to contact their counselors if they experience increases in relapse risk in between regularly scheduled sessions. However, such increases often come during evenings and weekends or when therapists are not available for other reasons.
- Procedures for marshaling other recovery supports are slow and cumbersome. Patients are urged to call peers in recovery and other supports when they feel at risk for relapse. However, patients may not have the necessary information when they most need it. They may also hesitate to reach out due to embarrassment or shame.

In the search for solutions to these challenges, digital interventions have become increasingly popular. Previous reviews of such interventions [25,26] reveal a large and complex picture with regard to targeted users, use, and efficacy. For example, these interventions have been developed for 3 distinct groups: those with hazardous alcohol or drug use, those currently in treatment for SUD, and those in recovery from SUD after undergoing treatment.

The concept “digital” also covers a variety of methodological strategies and elements. These interventions may contain a single element or a more complex collection of elements that build on digitized methods previously used in face-to-face interventions or methods uniquely developed for the current digital intervention. They may be meant to function as a stand-alone intervention or as an element in a larger intervention/support program. They may contain no interactive elements or different interactive elements in the form of automated responses or online real-time communication.

The technological solutions and platforms also vary. Some interventions are delivered on computer-based platforms (desktop or Web-based applications), while others are based on mobile phone technology platforms. Interventions feature a range of technological platforms, such as information websites or apps, assessment and monitoring technologies, automated or interactive voice response, text messaging, and chat rooms.

Papers on digital interventions for hazardous or risky alcohol use have been reviewed several times in the last 3 years, and papers on interventions for cannabis use were reviewed by Tait et al [27] in 2013. Dedert et al [26] describe these kinds of interventions as brief normative feedback on self-reported alcohol consumption, much in line with what is known as Screening and Brief Intervention (SBI). Dedert et al [26] found that the results of these kinds of interventions are much the same as found in nondigital SBI; a small short-term effect (consumption at 6 months follow-up) but no long-term effects. These results are confirmed in a newly published Cochrane review by Kaner et al [28]. In a meta-analytical comparison of digital and in-person delivered interventions, Cadigan et al [29] found no difference between the 2 modalities on short-term effect (less than 4 months), while the in-person interventions had stronger long-term effects. The same conclusion was drawn in the reviews conducted by Dotson et al [30] and Leeman et al [31], while Huh et al [32] even questioned the short-term effect of digital SBI. Tait et al [27], in their review of interventions for cannabis use, describe both the content of the interventions and the results regarding reduction in cannabis use, arriving at the same results as found in reviews of interventions for alcohol use.

In a review of 7 recent studies using more technologically developed program elements, Berman et al [33] were not able to find more positive results than in the traditional interventions. Interactive voice response interventions showed some short-term positive results on consumption, while text messaging and mobile phone apps showed no significant effects. A study by Cunningham et al [34] compared a brief with an extended intervention (AlcoholHelpCentre.net) and found that the extended intervention did not increase the effect. In recent studies, however, promising results have been obtained from adding new elements to standard interventions, such as gamification [35], booster email sessions [36,37], skills training via mobile phone apps [38], individually tailored text messaging [39], and Facebook delivery of personalized normative feedbacks [40].

Studies have also been done to evaluate digitized versions of existing interventions such as cognitive behavioral therapy (CBT) [41], motivational interviewing (MI) [42], and cognitive enhancement therapies such as cognitive training [43] or mentalization-based therapy [44]. None of the previous reviews on digital interventions report on results from these kinds of interventions, and it is outside the scope of this paper to do so. With the large number of interventions of this kind now developed and researched, such a review would be welcomed.

In this review, our prime interest is in new interventions developed specifically for digital platforms focused on supporting people in recovery from SUD by helping them achieve their substance use goals (eg, stay abstinent, using less or having a less damaging use pattern) and develop a more satisfying life situation. The review will cover the whole variety of interventions with regard to methodological strategies and technological solutions. In the review, we are interested in the feasibility of the interventions and effects on substance use and other aspects of recovery.

## Methods

### Search and Evaluation Criteria

In this review, our aim is to synthesize results on feasibility and effects found in primary study reports on digital interventions focused on supporting people in recovery from SUD. The concept “digital interventions” covers a range of intervention methods delivered through digital channels, and therefore a traditional meta-analytic review of the results of a specific method is not possible. However, a systematic approach was taken to identify relevant publications for our review of feasibility and effects. First, this review was based on a systematic search for studies in several databases using specific criteria on target groups, types of interventions, and outcomes. Second, the studies found in the search were systematically evaluated based on criteria relevant to the aims of the review. For example, in addition to reviewing quantifiable effects on substance use outcomes, we address feasibility features including patterns of use and user satisfaction. Third, all studies included in the review were systematically scored on specific features and effects of the interventions. The presentation and discussion of results is based on these scorings.

### Search and Exclusion History

Previous reviews on digital alcohol and drug interventions have shown that this is a large and complex research field, often with an imprecise use of concepts and descriptions regarding target groups, methods, and outcomes. In this review, we focus on digital interventions aimed at supporting a specific target: people with SUD who are working to achieve their long-term goals regarding substance use and the achievement of a more satisfying life through recovery.

To be sure that we did not exclude any relevant papers, we started with as wide a search strategy as possible. To this end, we adopted the strategy used by Dedert et al [26] as our starting point. These authors also conducted a comprehensive review, and their strategy is presented in detail in an appendix in their paper. Our first step was to implement the same search strategy as Dedert et al [26] and expand the search period to our current search date (November 2017). As they only searched for alcohol interventions, our next step was to conduct the same search, replacing the search term “alcohol” and its National Library of Medicine Medical Subject Headings terms with “drugs” and its Medical Subject Headings terms. The search was done in the following databases: MEDLINE, Embase, PsycInfo, and Cochrane Library. Together these searches (after excluding doublets) came up with a list of 1147 papers. The final step was to add additional papers from reference lists in the most recent of these papers. This resulted in adding 2 more papers.

We then started the process of excluding papers with no relevance to the aim of our review. This process was conducted in stages.

After a quick reading of titles and abstracts, the first author excluded 457 articles that were not about substance use/disorders and/or not about digital interventions. Based on a more thorough reading of the abstracts, the first author then excluded 265 articles found to be protocols, short intervention descriptions, editorials, notes, or comments.

Based on the title and a thorough reading of the abstracts, both authors cooperated in dividing the remaining 427 articles into 4 categories: (1) reviews on different kinds of digital interventions (n=21), (2) interventions aimed at reducing hazardous alcohol use (or, in a few cases, cannabis or other drug use) (n=269), (3) digitized versions of standard treatments such as CBT (n=94), and (4) unique digital interventions aimed at helping or supporting persons in recovery from SUD with or without co-occurring physical or psychiatric disorders (n=43).

The 43 articles on unique digital intervention were all read in full and scored by both authors. The scoring categories are shown in Multimedia Appendix 1 [45-86] and 2 [45-86]. Due to the wide range of retention, feasibility, and efficacy variables employed across the studies, it was not possible to score studies using the same set of variables. Therefore, outcomes were described in the appendices as reported in the articles reviewed. The lack of consistency across studies is most apparent in the data presented in the Retention/Feasibility column in the appendices. In the Effects column, findings from the main outcome variables of the studies are presented. Studies were categorized as positive when there was a statistically positive effect favoring the experimental intervention on at least one of the primary outcomes with no significant findings in the other direction on any other primary outcomes. Agreement between the 2 authors was very high with the few disagreements easily resolved through discussion. It is these 43 papers that form the basis for this review.

## Results

### Overview

The tables in Multimedia Appendix 1 present our review of 43 articles on 28 unique digital interventions meant to support people in recovery from SUD. Articles presenting different studies of the same unique intervention are grouped together for ease of interpretation of the full set of findings pertaining to each intervention.

The interventions are varied when it comes to methodological strategies and technological platforms and solutions. One of the primary distinctions pertains to their degree of complexity. We have chosen to divide the interventions into 2 categories based on complexity. Eighteen interventions are defined as simple in the sense that they consist only of 1 or 2 elements. They may contain only text messaging or only online counseling, or they may contain some sort of self-monitoring and brief feedback (text messaging or online counseling). Ten interventions are defined as complex in the sense that they consist of more than 2 elements. These digital support programs typically contain several functions.

In Multimedia Appendix 1, information is provided on the 3 other criteria we used to describe and categorize studies: age of participants, gender, and substance used. With regard to age, 65% (28/43) of articles focused on adults, 23% (10/43) on adolescents, and 12% (5/43) on both age groups. With regard to gender, 91% (39/43) studies included both men and women, 2% (1/43) included men only, and 7% (3/43) were unclear on the gender of participants. With regard to substance(s) used, 40% (17/43) studies focused on alcohol, 33% (14/43) on mixed substances, 9% (4/43) on alcohol and cannabis, 5% (2/43) on opioids, 7% (3/43) on stimulants, and 2% (1/43) on cannabis.

One article [45] reports on a survey to assess patient preferences on content in text messaging interventions but addresses no specific intervention. Another article [46] describes results from a survey about the general acceptance of different kinds of digital aftercare interventions among inpatients. These articles are therefore not included in the review of outcome effects.

### Publication Year

The rapid increase in development and research on the kind of digital interventions of interest to this review is clearly demonstrated when one looks at the publication year of the included articles. Even with the search spanning the 17 years, from 2000 to 2016, only 2 of the included articles were published before 2010, and 32 of the 43 included articles were published in the last 3 years, 2014 to 2017.

### Country

About half of the studied interventions are from the United States (16/28, 57%), 8 are from Europe (3 from Germany, 3 from Switzerland, 1 from Ireland, and 1 from Norway), 3 are from Australia, 1 is from Canada, and 1 is from Brazil.

### Types of Interventions

Only 6 of the 18 simple interventions were presented with a brand name. The most common element in these interventions was a 1-way or interactive text message service. One-way solutions typically consisted of a series of text messages with informative or supportive content delivered each day or less often for a fixed period of time. Interactive solutions typically contained standardized self-assessments of substance use, life situations, relapse risk factors, or medication compliance that were delivered as text messages and triggered automated responses or text messages from a counselor. Some simple interventions contained an online counseling service or online counseling in addition to monitoring.

Only 5 of the 18 simple interventions were meant to be stand-alone interventions (ie, without any other contact with the professional support system). All others were integrated in a larger support system that most often offered other kinds of face-to-face counseling or support services.

A clear majority of the simple interventions (12/18, 67%) were delivered on a mobile platform, and mobile phones are now the dominant device used in these kinds of interventions. Four interventions were delivered on a desktop or Web-based platform while 2 interventions used both platforms.

Nine of the 10 complex interventions were presented with a brand name. Six of the interventions are meant to be stand-alone interventions, while 4 are integrated with other treatment or aftercare services. Three of the interventions used a mobile platform such as a mobile phone, while 3 used a desktop or Web-based platform. Six interventions used a combination of desktop/website and mobile technologies.

The complex interventions contained a number of elements in different combinations. The most common features were systems for monitoring or check-ups, and some also included a Global Positioning System–based warning system. Other features included information and education modules; exercise modules for better concentration and relaxation; and modules to foster more effective coping strategies for harm avoidance, relapse prevention, and dealing with stress. Most of these interventions also contain interactivity modules such as feedback on monitoring results, delivery of supportive messages, online counseling and contact with peers, and chat rooms or digital self-help groups. Several programs have some kind of panic button, making it possible to reach counselors or peers in situations of urgent need for support.

Some of the interventions, such as Addiction Comprehensive Health Enhancement Support System (A-CHESS), have a clear theoretical foundation. A-CHESS is based on self-determination theory (SDT) and the relapse prevention model developed by Marlatt et al [87]. Consistent with SDT, the intervention program is designed to meet 3 fundamental needs: developing perceived competence, relatedness, and internal motivation. Consistent with the relapse prevention model, the program is meant to address and offer support in high-risk situations where relapse vulnerability is high.

Other interventions build on established nondigital support programs or borrow elements from treatment methods. Overcoming Addictions [70] builds on a support program developed and implemented by the Smart Recovery organization. Location-Based Monitoring and Intervention System for Alcohol Use Disorders (LBMI-A) is an example of an intervention borrowing principles or elements from different treatment methods, in this case CBT. Principles and elements from CBT and MI were also the basis for development of the Snow Control [83] and Can Reduce [84] interventions and the intervention (no brand name) studied by Tait [85,88]. However, these interventions are not strictly digitized versions of existing CBT or MI protocols; rather, they incorporate some of these features or elements within their own unique frameworks.

### Target Groups

Twelve of the simple interventions had adults as their target group, while 4 were intended for adolescents, and 2 did not discriminate their target group by age. None of the simple interventions discriminated their target group by gender, but it seems like 1 of the interventions only targeted men and it is uncertain if 1 other did the same. Nine of the simple interventions targeted people who had used or were using alcohol as their main substance, while 1 targeted stimulant users, 1 targeted cannabis users, 1 targeted opioid users (in maintenance treatment), 1 targeted alcohol and cannabis users, and 5 targeted those with mixed substance use patterns.

With regard to the complex interventions, 4 of 10 had adults as their target group while 2 had adolescents and 2 targeted both. Two of these interventions were not discriminating their target groups by age. None of these interventions discriminated their target group by gender, but 2 may have only reached men. Two of the interventions had people who had used or were using alcohol as their target group, while 2 were focused on cannabis users and 2 targeted both groups. Two interventions targeted stimulants users, and 1 addressed opioid users in maintenance treatment.

### Types of Studies

Fourteen of the 18 simple interventions had been studied with quantitative controlled methodologies while 3 had been studied quantitatively without control groups. In addition, 3 interventions had been studied with qualitative methodologies while 1 had been studied both quantitatively with a control group and qualitatively. One paper presented results from a survey on preferred text message content without referring to a specific intervention. The number of participants in the quantitative studies varied from 54 to 408, while the qualitative studies were smaller (eg, from 16 to 80 participants). The follow-up periods varied from 1 to 12 months.

Nine complex interventions had been studied with quantitative controlled methodologies and 1 quantitatively without a control group. The number of participants in these studies varied from 50 to 84, and the follow-ups varied from 2 to 12 months in duration. In addition, 2 complex interventions had been studied with mixed methods research designs and 1 intervention (A-CHESS) has been studied with several designs: controlled, uncontrolled, and mixed methods. These studies had from 29 to 349 participants and 2 to 12 months follow-up.

The last paper in Multimedia Appendix 1 was based on a survey of 374 inpatients about the feasibility of digitized aftercare interventions without referring to a specific intervention.

### Implementation

Overall, it appeared that the interventions were implemented successfully (without technical difficulties), although there was not a lot of information on this. The interventions were made available to eligible participants in the studies by forwarding links to internet sites or via mobile phone apps. The interventions in our review are generally not made accessible through an open app store download. A new commercial version of A-CHESS is, however, made available through the app stores. On the other hand, many recovery apps, not supported by research, are openly available in app stores. It is outside the scope of this review to make any evaluation of such interventions.

There was only 1 study focusing explicitly on prerequisites for a successful implementation of digital interventions. In a study on the implementation of A-CHESS, Ford [74] found that the following factors were important for a successful and sustained implementation of the intervention: strong leadership support, a staff that is passionate about the intervention, interpreting user feedback to re-engage users who had dropped out, including the intervention in meetings with staff and users, developing internal guidelines for using the intervention, and developing sustainable strategies for financing the intervention.

In 2 of the studies of complex interventions, participants were offered free phones and offered replacement phones if the first ones were lost, broken, or stolen. In the Check-In Program [79], 44% returned their first phone after the end of the 3-month study while 44% needed a replacement phone. In the first study of A-CHESS [76], 170 participants needed 116 replacement phones during the 8-month trial. In the other papers, there is no information about whether the participants were offered free phones or used their own phones.

### Rates of Sustained Use of the Interventions

The information in the papers on the simple interventions suggests that rates of use of the app or intervention were fairly uneven across the studies. If recruitment to the intervention (and study follow-up) took place through open websites, there was a large drop in the number of participants from those who accessed the site to those who registered in the intervention and those who accessed the first module (eg, first assessment) [51]. The same was seen in a study where possible participants were screened by general practitioners [54]. Among those who screened eligible for the intervention, only 50% accessed the first module and only 50% of those accessed the next module. Conversely, in studies where participants were recruited from patients in SUD treatment programs and where this information is reported, the rate of sustained use of the intervention seems to have been as high as 75% at the end of the study [50].

In the complex interventions, rates of use seemed to be fairly high at the beginning, typically around 90% in the first few weeks. But rates of use of the interventions dropped very quickly; for example, from a mean of 7.3 to a mean of 1.3 log-ins each week during the first 3 months in the intervention studied by Campbell [70] or to 18% after 6 weeks in the interventions studied by Schaub [83,89]. Two interventions had higher rates of use; 1 reported a drop from 63% completing the first module to 48% completing the last module during the 3-week intervention [85] and a second reported that on average the participants completed 7 of the 8 sessions in the intervention [57]. Two of the complex interventions seem to have high overall rates of use. In the My First Year of Recovery (MyFYR) intervention [82], 78% completed the 1-year-long program and 70% of those who relapsed during the intervention remained engaged or re-engaged and were able to complete the intervention. In the case of the A-CHESS intervention, 78% of the sample was still using the intervention after 4 months [71]. The intervention Check-In Program was combined with a computer-based psychoeducative program (Therapeutic Education System) in one of the study conditions [79]. In the combined condition, the retention rate was 84% by the end of the study, compared to 56% in the uncombined condition (*P*=.031).

### Intensity and Duration of the Interventions

The simple interventions varied quite a bit in intensity and duration. The most intense intervention was studied by Reback et al [65]. In this intervention, the participants received on average about 10 messages each day and sent as many replies over the 2-month duration. The participants received additional support feedback in response to about a third of their messages. The shortest intervention was 2 weeks in duration [64]. The intensity was also very high in this intervention; on average the participants received 8 and sent 4 messages each day. The rest of the interventions were less intense and had a longer duration. In these, participants typically received and sent 2 messages each day for 3 months [48] or 10 months [63], 1 message each day for 2 to 4 months [52,55,58,67,68], or 1 each week for 6 months [62]. There is not much information in the papers documenting whether the participants actually read the messages they received. There are 2 papers reporting on this; in Agyapong [49], participants read 67% of the received messages, and in Haug [90], participants responded to 88% of the messages.

The complex interventions were generally more intense than most of the simple interventions. In most interventions, it was possible for participants to log in to several elements or modules each day, making the interventions more or less intense based on how many modules the participants accessed each day. In studies of the complex interventions, the duration of the interventions varied between 2 and 12 months.

### Intervention Content and Use of Features

The text messages in the simple interventions covered a large range of topics. One article [49] reported on which topics were of greatest interest to the participants. Among those were messages on motivation for recovery and relapse prevention and reminders on why and how to stay abstinent. The same kinds of topics were recommended by participants in Gonzales [78] and in the survey by Tofighi [45].

Use of different kinds of modules varied between interventions and during the intervention in the 3 complex interventions for which information on this was provided. None of the participants in the LBMI-A intervention [78] used the skill modules for resisting urges to drink or drink refusals and very few used the psychoeducational modules after week 2 of the intervention, while most of the participants continued using the monitoring modules. The 25 participants in the experimental condition of the study of the Check-In Program [79] completed, on average, 21 self-management modules and 9 functional analysis modules during the 3-month intervention. Several of the studies on A-CHESS present information on the use of the different modules of the intervention over time. The study by Dennis [73] showed that adolescent participants using the intervention completed 89% of the assessments in the ecological momentary assessment module and accessed the ecological momentary intervention module 78% of the days of intervention. The most used ecological momentary interventions were recovery support, motivation, relaxation, and social networking. In Gustafson [76], it was reported that the participants, on average, used the intervention 41 days during the 8-month trial and that 72% of the participant pressed the panic button at least once. McTavish [77] presents the use of different modules of the intervention during the first 4 months of the first trial and relates it to the theoretical principles of the intervention (SDT). McTavish [77] found that the percentage of participants using the intervention dropped from 94% the first week to 78% the fourth month. Use of modules related to perceived competence dropped from 80% to 39%, modules related to autonomous motivation from 84% to 66%, and modules aimed at increasing the feeling of relatedness from 91% to 76%.

### User Satisfaction

The articles reported high satisfaction with the simple interventions. For example, some studies reported that the participants were generally highly satisfied [49,56] or that they “felt connected” via the intervention [55]. Bradford [56], Gagnon [58], and Ingersoll [63] reported that 80% to 90% of participants were satisfied, finding the interventions easy to use and being confident and comfortable in using them, while Gonzales [60] found that 70% were positive about the intervention (20% were ambivalent and 10% negative). In the intervention studied by Haug [90], the overall satisfaction was a bit lower: 63% of participants found the intervention generally helpful, but 75% wanted to do the program again, which suggests a somewhat higher level of satisfaction.

Three of the papers reported on the participants’ expressed satisfaction and the usefulness of the complex interventions. Guarino [79] reported that participants found the Check-In Program intervention highly acceptable and useful (75 to 80 points on a 100-point scale). Hasin [80] reported that 80% or more of the participants in the Health Call intervention gave very positive feedback on user interface and satisfaction with the content. Campbell [91] reported that the participants in the Overcoming Addictions intervention found the social support and awareness reminders to be the most helpful element.

### Intervention Effects on Substance Use Outcomes

Of the 24 studies of 18 simple interventions included in this review, 7 featured a control condition and produced positive effects on substance use outcomes. These were a stepped care intervention that included computerized feedback [92], My Assessment by Bradford et al [56], 2 HIV risk reduction interventions [58,65], ESQYIR by Gonzales et al [61,93], an in-home messaging device by Santa Anna et al [67], and an internet-based relapse prevention program [69]. Three of these interventions were delivered by text messaging [59,61,65]. Other positive interventions consisted of an integrated psychosocial assessment delivered through an app [56], a website that provided tailored audiovisual messages regarding safer injection practices [58], and 20 online lessons that provided information on addiction and relapse prevention skills for adolescents [69].

Conversely, 8 studies of simple interventions with control conditions found no positive effects on substance use outcomes. The interventions tested in these studies were a text messaging system [47,48], integrated online counseling intervention [50], computerized intervention for anger management [57], integrated text messaging systems that included online counseling [62-64], and a text messaging intervention that included medication monitoring and support [94]. Another 9 studies either did not include a control condition or did not examine substance use outcomes. By a simple box score calculation, these results indicate that 7 of 15 studies with control conditions (47%) produced positive effects on substance use outcomes. When considered at the level of the interventions, 7 produced positive results in at least 1 study, whereas 7 produced negative results in 1 or more studies with no positive results in other studies (ie, 50% of interventions positive).

The interventions in the 7 positive studies all had moderate effect size advantages over the control conditions on 1 of the primary outcomes. The control conditions were bona fide interventions, usually treatment as usual without the digital component, except in the Bischof study [92], which employed an untreated control condition, and Trudeau et al [69], which used a wait list control. Five of the interventions addressed drugs, and 2 focused on alcohol. Five interventions were integrated, while 2 were stand-alone. The studies with interventions that did not produce positive effects over control groups look similar to those that did on strength of the control groups, targeted substance, and stand-alone versus integrated format. All of the negative studies included bona fide active control conditions, primarily behavioral treatment as usual. Three studies focused on alcohol only, while 4 addressed mixed or poly substance use. Finally, most of the interventions were integrated, with 2 stand-alone. It should be noted that 2 studies were likely underpowered [50,64], as they produced positive but nonsignificant effects on primary SUD outcomes.

The 10 complex interventions were evaluated in a total of 19 publications included in the review. The A-CHESS intervention was studied in 7 reports; only 2 other interventions were examined in more than 1 report [85,88]. The Hasin et al [80] and Aharonovich et al [81] publications were of the same intervention in 2 separate studies, whereas the 2 Tait et al [85,88] publications reported results from different follow-ups in the same study. Of the studies included, 9 yielded positive results, 5 produced negative results, 2 did not include control conditions, and 3 did not examine SUD outcomes. Four of the 9 positive studies were of the A-CHESS system. In the one large scale A-CHESS randomized controlled trial, those randomized to A-CHESS reported fewer heavy drinking days over a 12-month follow-up than those who did not receive A-CHESS: 1.39 versus 2.75 out of the prior 30 days [76]. A second study showed that ecological momentary assessment data gathered on A-CHESS could predict upcoming relapse episodes [71]. A third study indicated that adolescents who accessed 2 or more supportive functions on A-CHESS within 1 hour after reporting elevated relapse risk were less likely to go on to relapse in the next 7 days that those who used fewer supportive A-CHESS functions [73]. It should be noted that the positive results in this paper could have simply reflected self-selection, with more motivated participants both accessing A-CHESS more frequently and having better outcomes. Finally, a fourth study found that the effects of A-CHESS on the risky drinking days outcome was mediated by participation in outpatient SUD treatment [75].

Other complex interventions that generated SUD outcomes superior to comparison conditions were a mobile phone–delivered CBT-like intervention that consisted of 7 modules [78], a mobile phone–based monitoring program (HealthCall) that graphs results and arranges for contact with a counselor [80], a mobile phone–based treatment extender compatible with the computerized Therapeutic Education System [79], a Web-based self-help intervention that included chat counseling [95], and a Web-based intervention that included self-monitoring and weekly feedback from counselors [86].

Complex interventions that did not produce positive effects on SUD outcomes were a Web application based on Smart Recovery [70], a study of A-CHESS where there was no difference in A-CHESS use between lapsers and nonlapsers [71], an initial pilot study of HealthCall [80], an 8-module Web-delivered self-help intervention based on CBT and MI [96], and a Web intervention based on CBT, MI, and harm avoidance approaches [85,88]. According to a simple box score calculation, these results indicate that 9 of 14 controlled studies (64%) produced positive effects on substance use outcomes. When considered at the level of the interventions, 6 produced positive results in at least 1 study, whereas 3 produced negative results in 1 or more studies with no positive results in other studies (67% of interventions positive). There was 1 negative A-CHESS study but 4 positive ones, and 1 negative study of Healthcall but 1 positive one.

In the studies with positive effects, 6 featured integrated interventions and 3 stand-alone interventions. Four of the studies focused on alcohol, 2 on cannabis, 1 on both alcohol and cannabis, and 2 on mixed substance use. Four of the studies featured no treatment or waitlist control conditions, 1 included another online intervention, 1 included an active behavioral intervention control condition, and 1 had a standard methadone maintenance control condition (2 studies did not include a treatment control condition but rather focused on the ability of the A-CHESS system to predict relapse and deliver just-in-time interventions). Effect sizes were generally in the moderate range, although the major A-CHESS trial [76] produced a smaller effect (*d*=.18-.25) and the Tossman et al [86] study produced a large effect (*d*=.75). In the studies that did not produce positive treatment effects, 2 interventions were integrated and 3 were stand-alone. Two studies focused on alcohol, 1 on cannabis, 1 on alcohol and cannabis, and 1 on stimulants. The control conditions were generally fairly weak, including a waitlist control, psychoeducation, historical interactive voice response intervention group, and Smart Recovery.

### Intervention Effects on Other Outcomes

There was not much information in the papers on effects on outcomes other than substance use. Four of the papers on simple interventions reported on changes in use of other services. Gonzales [61] reported significantly higher attendance at self-help meetings and recovery-oriented activities among those in the experimental condition compared to controls, and Ingersoll [63] reported that adherence to antiretroviral treatment increased by 19 percentage points in the experimental condition compared to a 9 percentage point increase in the treatment as usual condition. Bischof [92] reported a drop in face-to-face counseling time of 50% in the experimental condition, while Lucht [64] reported that the participants in the experimental condition spent significantly more days than the controls in psychiatric hospital. While the results in the first 3 of these articles may be evaluated as positive results, we are not sure that the result reported by Lucht could be evaluated in this way. Only 1 of the articles on the complex interventions reported on changes in use of other services. Tait [97] reported that those in the experimental condition significantly increased their general help seeking compared to controls.

Three of the articles on simple interventions also reported on other outcomes. Cougle et al [57] reported that hostile interpretation training led to greater improvements in interpretation bias, trait anger, and anger expression. Reback et al [65] reported that the participants in the experimental condition significantly reduced their risky sexual behavior, while Rooke [66] reported a significant reduction in depression in those in the experimental condition compared to controls. Four of the articles on complex interventions also reported some information on effects on outcomes other than substance use. Gustafson [76] found that A-CHESS had no impact on negative consequences of drinking, Glass et al [75] reported that A-CHESS increased participation in outpatient treatment following rehabilitation, Schaub [95] found that Can Reduce did not affect mental health measures, and Tait [97] found that the intervention they studied made no difference on psychological distress. On the other hand, Tait found that the intervention led to a significant reduction in days of general impairment.

## Discussion

### Principal Findings

The development and evaluation of digital interventions aimed at preventing or treating substance use–related problems and disorders is a rapidly growing field. A large number of articles were identified on this topic, and most of these reports focus on interventions developed and studied in the last few years. The concept of digital interventions includes such a wide variety of interventions with regard to aims, target groups, methods, and technological solutions that it is impossible to cover them all in one review. In this review, we therefore focused more narrowly on unique interventions aimed at supporting people in recovery from SUD. But as we did not want to miss any studies due to imprecise use of concepts in relevant studies, we started out with a wide collection of search terms. We found a large number of papers on interventions for hazardous or risky drinking and digital interventions in the SUD field that were not relevant to the aim of this review. However, including them in the first stage of the evaluation process gave us the opportunity to suggest a categorization of interventions, created specifically for digital platforms, that may become useful in further development, research, and review of such interventions.

Although not the focus of this review, it is clear that the field of digital interventions aimed at hazardous but not disorder-level alcohol or drug use is a large and well-reviewed area but with relatively modest results. Digitizing existing treatment such as CBT has also become an important and promising area, but to our knowledge without any systematic review done so far. Our quick reading of these studies gave us an impression that these kinds of interventions could make an important contribution to the development of more available and effective treatment of SUD.

The 43 articles that reported on the studies that were evaluated as relevant to the aim of this review seemed to cover a larger variety of interventions then the 235 and 87 articles in the other categories. This made it important to define scoring criteria so as to conduct a review as systematically as possible. Two of these criteria—integrated versus stand-alone in relation to other services and substances of abuse—did not appear to have a significant impact on the feasibility or efficacy of the interventions. There was insufficient variation in 2 of our other criterion—gender and age of the participants—to draw any conclusions regarding their impact on outcomes. Finally, the categorization of the interventions that was based on their complexity (number of elements or modules) did appear to make a difference on both feasibility and effects on substance use. However, the more positive substance use outcomes for the complex interventions may have been due to differences in the strength of the control conditions used in these studies, which was another criterion examined in the review.

### Feasibility Strengths and Weaknesses

International market figures show that smartphones have become the dominant digital device in the Western world, with an 80% to 90% share of the total mobile phone market, and are quickly also becoming the dominant device in the rest of the world, passing 50% of the market [98]. Mobile phones are also quickly replacing other digital devices, such as laptops and tablets, as the device most likely to be used daily. It is therefore understandable that mobile phones are becoming the dominant technological platform for digital interventions, making it even more appealing as a tool for offering effective and flexible solutions for recovery support. The simple interventions seemed relatively easy to develop and implement, especially those using standard text messaging and mobile phone apps. Complex interventions seemed also relatively easy to implement, but the study by Ford [74] showed that there are many organizational prerequisites to achieving a sustainable implementation of such interventions over time. In addition to addressing questions about the feasibility of various technical solutions, it is important to determine whether the organizational prerequisites are in place to sustain the implementation over time before implementing a digital intervention.

Another challenge in the implementation of digital interventions is, of course, that the participants need the required technical equipment. The initial cost of buying such equipment may be too high for many people with SUD, and mobile phones may easily be broken, lost, or stolen. Buying and replacing phones for participants may be a solution in a research project but appears not to be a sustainable solution in real-life contexts. Our impression is that owning a mobile phone, which increasingly means a smartphone, is regarded by people in recovery from SUD as highly desired and even viewed as an essential expense. Smartphones are not only replacing ordinary mobile phones but also personal computers. This means that interventions have to be built on mobile platforms, using the flexibility and technological possibilities of modern smartphones. But developers of digital interventions also need to adjust their methodological and design strategies to the particularities of modern mobile phone technology and user interface designs [99]. Interventions based on traditional desktop, website, or mobile phone technologies may already be out of date, as they may not contain the functionalities and user interface designs required to reach potential intervention participants and keep them engaged with the program.

Rates of sustained use of the apps and interventions varied to a considerable amount. Access to simple interventions via websites or mobile phone app stores made it possible to reach many potential participants, but retention in most interventions dropped quickly. This is a method often used in interventions aiming at hazardous but not disorder-level substance use. However, if the goal is to offer recovery support, it appears to be more effective to recruit people in treatment or self-help group/network settings to both reach the most relevant participants and keep rates of intervention use high over time.

In the complex interventions, retention was generally high both in the beginning and over time. Here it seems that the challenge was more that the frequency of use was very different for different kinds of elements or modules. Generally, the information and educational modules appeared to be most frequently used in the beginning of the intervention, while modules supporting continuous monitoring and communication with counselors and peers retained higher rates of use over time. Instead of evaluating this as a weakness of the intervention program, these findings could lead to the development of interventions in which different kinds of modules are presented to the participants in a planned “tunneled” sequence [100]. Or it might be advantageous to divide some interventions into separate modules, making it possible to directly access each module.

### Effects

In formulating the aim of this review, we were interested in how digital interventions could support people in recovery from SUD with regard to their goals for achieving abstinence or reduced substance use as well as better health and life situations and use of other services. The review showed, however, that few studies reported on anything other than changes in substance use.

Across simple and complex interventions, slightly more than half (55%) of the studies with control conditions generated positive findings on 1 or more substance use outcomes, with 57% of the interventions also found to be efficacious in 1 or more studies. In the positive studies, effects were typically in the small to moderate range, with a few studies yielding larger effects. At first glance, the simple interventions appeared to be somewhat less effective than the complex ones. In studies of simple interventions that employed control groups, 47% yielded positive findings on 1 or more of the primary substance use outcomes, with 50% of the interventions producing positive results in at least 1 study. Studies testing complex interventions, on the other hand, generated positive effects in 64% of studies with control groups, with 67% of the interventions producing positive effects in 1 or more studies. However, studies of simple interventions were more likely to include stronger control conditions than studies of complex interventions. As was noted earlier, this might explain why simple interventions were less likely to produce positive effects. In addition, 2 studies of simple interventions appeared to be underpowered and might have shown positive effects with larger samples. It did not appear that the substance targeted in the study or whether the intervention featured a stand-alone versus integrated format accounted for the results.

Overall, these results do not provide consistent, strong support for the efficacy of these interventions. However, the heterogeneity in results, with some interventions appearing to be more promising than others, indicates that more work is needed to better understand the characteristics of efficacious digital recovery support interventions. Further research should also shed light on the kinds of individuals most likely to benefit from different digital interventions and at what points in their recoveries the largest effects are obtained.

In the scoring of the studies, we systematically searched for other outcomes than changes in substance use such as psychological health, medication, housing, employment, social functioning, and criminality. But as the articles in this review contained little information on such effects, it is not possible to draw strong conclusions on this issue. We have, however, the impression that the interventions had no or only modest effects on such outcomes. As recovery should focus on many more issues than just changes in substance use, it is a weakness in the studies that they did not focus more on such outcomes and a weakness in the interventions that they either are not aiming at contributing to such changes or that they have no effect when they try to do so.

### Conclusions

The digital interventions included in this review are in general feasible but are not consistently effective in helping people in recovery from SUD reduce their substance use. It is questionable whether they are effective in supporting people to achieve other recovery goals, given the relative lack of information on this in the studies. Mobile phones appear to be the most feasible technological platform for such interventions. Single interventions, such as 1-way or interactive text messaging or text messaging in combination with a simple monitoring module are relatively easy to develop, implement, and sustain and can be an effective supplement in continuing care and support programs. Complex interventions appear to be feasible and some of them are also modestly effective. They require, however, more technological and organizational resources to develop, implement, and sustain. It also appears that they could benefit from being developed into more sequentially and individually tunneled programs or being divided into single, directly accessible interventions.

Participants’ general satisfaction with the studied interventions should be regarded as the best inspiration to develop even more feasible and effective digital interventions, using all the technological possibilities and appealing user interface designs of modern mobile technologies. However, these technological solutions are only relevant if they are adjusted to the life situation of potential users and the organizational and knowledge-based framework of the support systems they are meant to be a part of and they make a difference in helping participants reach their recovery goals.

## Appendix

Multimedia Appendix 1 Intervention and study characteristics.

Multimedia Appendix 2 Intervention retention, feasibility, and effects.

## Abbreviations

A-CHESS: Addiction Comprehensive Health Enhancement Support System
CBT: cognitive behavioral therapy
LBMI-A: Location-Based Monitoring and Intervention System for Alcohol Use Disorders
MyFYR: My First Year of Recovery
MI: motivational interviewing
SBI: Screening and Brief Interaction
SDT: self-determination theory
SUD: substance use disorders
