# Supplementary material for: Feasibility and Effects of Digital Interventions to Support People in Recovery From Substance Use Disorders: Systematic Review
Source: J Med Internet Res. 2018 Aug 23;20(8):e255. doi: 10.2196/jmir.9873 (PMC6127498; doi:10.2196/jmir.9873)
Supplement: Multimedia Appendix 1 [file jmir_v20i8e255_app1.pdf]

Multimedia Appendix 1. Intervention and study characteristics.

**Intervention brand name** (First author if no brand) References	**Simple / Complex**	**Country**	**Type of intervention**	**Targeted groups: Age Gender Substance**	**Type of study: Design N Follow up**
Agyapong [47-49]	Simple	Ireland	Stand alone, text messaging, mobile platform	Adults<br>Men and women<br>Alcohol	Quant/ control<br>N=54<br>3 months
Alemi [50]	Simple	USA	Integrated, online counseling, desktop and mobile platforms	Adults<br>Men and women<br>Mixed substances	Quant/<br>no control<br>N=79<br>4 months
Andrade [51]	Simple	Brazil	Stand alone, online counseling with CBT elements and drinking monitoring, mobile platform	Adults<br>Men and women<br>Alcohol	Quant/<br>no control<br>N=312<br>1 month
Aschbrenner [52, 53]	Simple	USA	Integrated, text messaging and monitoring (medication compliance) and daily support (coping strategies), mobile platform	Adults<br>Men and women<br>Alcohol	Qualitative<br>N=17<br>3 months
Bischof [54]	Simple	Germany	Integrated, stepped care with computerized feedback on monitored stage of change (TTM), face to face counseling and follow up telephone counseling, mobile/ desktop platform	All ages<br>Men and women<br>Alcohol	Quant/ control<br>N=408<br>12 months
Bjerke [55]	Simple	Norway	Integrated, text messaging, mobile platform	Adults<br>Men and women<br>Mixed substances	Qualitative<br>N=16<br>4 months
**My Assessment** [56]	Simple	Australia	Integrated, electronic psychosocial assessment, mobile platform	Adolescents<br>Men and women<br>Mixed substances	Quant/ control<br>N=339<br>6 months
**IBM-H** (Interpre-tation Bias Modification-Hostility) [57]	Simple	USA	Stand alone, 8 session intervention designed to modify interpretations of social situations to reduce hostility vs. videos on healthy habits and self-care, web platform.	Adults<br>Men and women<br>Alcohol	Quant/ control<br>N=58<br>2 months
Gagnon [58]	Simple	Canada	Integrated, tailored audiovisual messages (safer injection), web platform	Adults<br>Men and women<br>Opioids	Quant/ control<br>N=260<br>3 months
**ESQYIR** (Educating & Supporting inquisitive Youth in Recovery) [59-61]	Simple	USA	Stand alone, text messaging (including self-monitoring, support), mobile platform	Adolescents<br>Men and women<br>Mixed substances	Quant/ control and qual<br>N=80<br>9 months
Haug [62]	Simple	Switzerl.	Integrated, text messaging (including monitoring, support, online counseling), mobile platform	Adults<br>Men and women<br>Alcohol and cannabis	Mixed methods<br>N=50<br>6 months
Ingersoll [63]	Simple	USA	Integrated, text messaging (including monitoring, counseling), mobile platform	Adults<br>Men and women<br>Mixed substances	Quant/ control<br>N=63<br>6 months
Lucht [64]	Simple	Germany	Integrated, text messaging (including monitoring, telephone support), mobile platform	Adults<br>Men and women<br>Alcohol	Quant/ control<br>N=80<br>2 months

Reback [65]	Simple	USA	Stand alone. Theory- based Health Belief Model (HBM), Social Cognitive Theory (SCT), and Social Support Theory (SST) SMS conditions mobile platform	Adults<br>Men<br>Stimulants	Quant/ control but no randomization<br>N=52<br>1 month
Rooke [66]	Simple	Australia	Stand alone, described only as a “smart phone app” with no additional information provided, mobile platform	Adolescents<br>Men and women<br>Cannabis	Quant/<br>no control<br>N=111<br>2 months
**IHMD** (In-Home-Messaging-Device) [67]	Simple	USA	Integrated, text messaging (including monitoring, skills development), mobile platform	Adults<br>Uncertain gender<br>Alcohol	Quant/ control<br>N=121<br>3 months
**MEMS** [68]	Simple	USA	Integrated, text messaging (including medication monitoring, support, reminders), mobile platform	Adults<br>Men and women<br>Alcohol	Quant/ control<br>N=76<br>2 months
**Navigating my Journey** [69]	Simple	USA	Integrated online relapse prevention supplement for adolescents in SUD treatment. Based on MET/CBT, compared to attention control	Adolescents<br>Men and women<br>Mixed	Quant/ control<br>N=129<br>6 months
**No intervention** [45]	Simple	USA	Text messaging content to improve medication compliance, but no specific intervention	Adults<br>Men and women<br>Opioids	Survey<br>N=97<br>NA
**Overcoming Addictions** [70]	Complex	USA	Stand alone, self-help program consisting of the four modules in the Smart Recovery program (education and exercises) and additional information links/pop-ups, desktop platform	Adults<br>Men and women<br>Alcohol	Quant/ control<br>N=189<br>6 months
**A-CHESS** [71-77]	Complex	USA	Integrated, the alcohol version of more generic program consisting of monitoring, information, support and interactivity with counselors and peers, mobile platform	All ages<br>Men and women<br>Alcohol;<br>alcohol and cannabis	Quant/ control;<br>quant/no control;<br>mixed<br>N=29-349<br>up to 12 months
**LBMI-A** (Location-Based Monitoring/ Intervention for Alcohol Use Disorders [78]	Complex	USA	Stand alone, CBT-inspired program with seven modules consisting of psycho-educational material and tools, and monitoring, mobile/ desktop platform	Adolescents and adults<br>Men and women<br>Alcohol and cannabis	Quant/ control<br>N=54<br>6 weeks
**Check-In Program** [79]	Complex	USA	Integrated, two skill-based interactive modules: “functional analysis” and “self-management”, mobile platform	Adults<br>Men and women<br>Opioids and mixed substances	Mixed methods<br>N=50<br>3 months
**Health Call** [80, 81]	Complex	USA	Integrated, monitoring program based on 30 daily questions, offers contact with counselor, mobile platform with smartphone<br>A second study compared Health Call plus MI to MI only	Adolescents<br>Men and women<br>Alcohol and cannabis<br>Adults<br>Men and women<br>Mixed substances	Mixed methods<br>N=84<br>2 months<br><br>Mixed methods<br>N=47<br>2 months
**MyFYR** (My First	Complex	USA	Integrated, information, monitoring, care management, support, self-help	Adults<br>Men and women	Quant/no control

Year in Recovery) [82]			groups, mobile/ desktop platform	Alcohol and mixed substances	N=198 12 months
**Snow Control** [83]	Complex	Switzerl.	Stand alone, eight CBT, MI, self-control based, interactive modules, mobile/desktop platform	Adults Men and women Stimulants	Quant/ control N=196 6 months
**Can Reduce** [84]	Complex	Switzerl.	Stand alone, same as Snow Control but adjusted to cannabis users, one study arm with chat room added, mobile/desktop platform	Adolescents and adults Uncertain gender Cannabis	Quant/ control N=308 3 months
Tait [85]	Complex	Australia	Stand alone, CBT, MI, harm avoidance based, automated, self-guided sequential modules, desktop platform	Adolescents Uncertain gender Stimulants	Quant/ control N=160 6 months
**Quit the Shit** [86]	Complex	Germany	Stand alone, self-regulation/control based, four phases, self-monitor, weekly feedback/chat, desktop platform	Adolescents and adults Men and women Cannabis	Quant/ control N=206 2 months
**No intervention** [46]	Complex	Germany	Acceptance of digital after care programs, not only SUD, no specific intervention	Adolescents and adults men and women NA	Survey N=374 NA
