# Supplementary material for: Feasibility and Effects of Digital Interventions to Support People in Recovery From Substance Use Disorders: Systematic Review
Source: J Med Internet Res. 2018 Aug 23;20(8):e255. doi: 10.2196/jmir.9873 (PMC6127498; doi:10.2196/jmir.9873)
Supplement: Multimedia Appendix 2 [file jmir_v20i8e255_app2.pdf]

Multimedia Appendix 2. Intervention retention, feasibility and effects.

**Intervention brand name** (First author if no brand) References	**Simple / Complex**	**Retention (in intervention) Feasibility**	**Effects (changes in substance use / other effects)**
Agyapong [47-49]	Simple	No tech implementation problems, few drop-outs from the intervention, high satisfaction, 67% read all texts, highest interest in texts regarding motivations for recovery, relapse prevention and reminders on abstinence	Agyapong et al. (2012): EXP=CTR on CAD: 88.3 (sd=6.2) vs. 79.3 (sd=6.2); p= .08<br>Agyapong et al. (2013a): EXP=CTR on CAD<br>Agyapong et al. (2013b): NA
Alemi [50]	Simple	Not reached: 26%,<br>irregular contact: 31%	EXP=CTR on rates of drug use
Andrade [51]	Simple	Number of participants:<br>-accessing the application: 32000<br>-registered into the intervention: 3389<br>-conducted first assessment (AUDIT): 929	< 50% follow-up rate in study. Pre-Post reductions in alcohol consumption were 44% in high risk drinkers, and 58% in possibly dependent drinkers
Aschbrenner [52, 53]	Simple	NA	Aschbrenner et al. (2016): No SUD themes emerged in analysis of text messages<br>Ben-Zeev et al. (2014): NA<br>Other themes emerging in messages: mental health symptoms, coping strategies, treatment and management, motivation, goal-setting, independent living, lifestyle, social relationships, leisure activities
Bischof [54]	Simple	Drop out from intervention:<br>-after screening positive for alcohol problems: 50%<br>-after first phase of the stepped care: 50%	Stepped care performed as well or better than standard care on all drinking outcomes. Grams alcohol per day: SC= 33.2, FC= 38.3, p= .03. Both were better than control on some secondary outcomes, including reductions in grams of alcohol and in binge drinking in participants classified as alcohol abuse/at risk drinkers at baseline.<br>Other: Stepped care resulted in 50% drop in counseling time (p=0.001)
Bjerke [55]	Simple	Eight patients sent 98 texts during 4 months and eight counselors sent 112 texts; patients reported “feeling connected” to the support system	NA
**My Assessment** [56]	Simple	Very high satisfaction with the intervention:<br>-easy to use: 92%<br>-confident it provides an accurate picture of the user: 74%<br>-comfortable using it: 77%	Increased reporting of socially undesirable behaviors (e.g., alcohol, drug, nicotine use; sex; non-heterosexual orientation; having sex without a condom, having STI check; self-harmed; putting self in unsafe situation) at rates 2.8 to 10.4 times higher than interview approach. Effect sizes .15 to .38.
**IBM-H** (Interpre-tation Bias Modification-Hostility) [57]	Simple	Participants completed 7.7 of 8 sessions	No condition differences on drinking outcomes. IBM-H produced greater improvements in interpretation bias, trait anger, and anger expression than control.
Gagnon [58]	Simple	No fear of accessing the intervention, capable of using it, only brief messages feasible as the users are in a hurry to inject	Intervention produced lower rates of use of dirty needles at 1 month: RR= 0.47, p= .004. Effect not present at 3 months.
**ESQYIR**	Simple	Reaction to intervention: positive= 70%;	Gonzales et al. (2014a): Text condition produced

(Educating & Supporting inquisitive Youth in Recovery) [59-61]		ambivalent= 20%; negative= 10%,<br>Most recommended themes:<br>-positive appraisal: 90%<br>-change tips: 85%<br>-motivation reinforcement: 80%<br>-coping strategies: 75%<br>Recommendations:<br>-frequency: once a day (late afternoon/evening)<br>-known sender (peers)<br>-intervention should last for 3 months	greater reduction in substance use severity than control (treatment effect by time interaction p= .03), Relapsed after 90 days: EXP=21.4% vs Control=59.3%.<br>Gonzales et al. (2016): Text condition produced lower relapse rates at 6 months (OR= 0.72, p= .02) and 9 months (OR= 0.74, p= .01), relapsed after 6/9 months: (28.6/14.7 vs. 54.1/42.9)<br>Gonzales et al. (2014b): NA<br>Other: Significantly higher attendance in self-help meetings and participation in recovery-oriented activities in text condition
Haug [62]	Simple	Response rate to messages: 88%<br>Sent at least one call-for-help reply: 44%<br>Satisfaction:<br>-helpful (in general): 63%<br>-helpful in adhering to personal drinking goal: 56%<br>-do the program again: 75%	Rate of at risk alcohol use was 41.7% in control and 28.6% in text message intervention group (NS)
Ingersoll [63]	Simple	Satisfaction with intervention:<br>-4.7 of 5 on most variables, but text message group not much better than TAU.	No difference on days of substance use at post treatment or 3 month follow-up. Both conditions reduced days of use by about 18 percentage points.<br>Other: Antiretroviral treatment adherence increased 19% in text vs 9% in TAU (p=0.05)
Lucht [64]	Simple	Successful technical implementation and rapid inclusion of users.<br>672 messages to patients, 690 replies from patients (10% SOS calls),<br>252 supportive feedback messages, 1202 emails from system to therapist.<br>General high satisfaction with intervention	Rates of low risk consumption at end of trial were 55.7% in SMS group vs. 40% in TAU (p= .12).<br>Patients in SMS group had more days of inpatient detox and addiction treatment days than TAU.<br>Other outcomes: SMS group more days in psychiatric hospital than TAU
Reback [65]	Simple	On average participants received 8 messages and sent 4 messages to staff each day, during the two-week long intervention	Increased exposure to HBM or SCT based messages relative to other types of messages was associated with decrease in frequency of methamphetamine use (IRR= 0.60).<br>Other outcomes: HBM and SCT significant reduction in risky sexual behavior
Rooke [66]	Simple	NA	In an uncontrolled study, reductions were observed on days of cannabis use, cannabis related problems, and self-efficacy to resist cannabis after one month of access to app.<br>Other: sign red. in depression after one month
**IHMD** (In-Home-Messaging-Device) [67]	Simple	NA	At one month, vets in IHMD drank on fewer days (p= .02) and had fewer binge drinking days (P<.0001). At 3 months, no difference on drinking days but continued advantage on binge drinking days (p< .001).
**MEMS** [68]	Simple	Control and intervention participants: No difference on number of adherence reminders over a 56 day study	Naltrexone compliance was higher in EXP than CTR at mid study, but not at end. No difference between conditions on alcohol craving or alcohol use
**Navigating my Journey** [69]	Simple	NA	Experimental condition produced greater decreases in drug use at 3 but not 6 months, and greater increases in motivation to change at 3 and 6 months. No intervention effects on self-efficacy, relapse coping skills, or alcohol use.
**No intervention** [45]	Simple	Interested in receiving messages: 90%<br>Highest interest in the beginning and after ending treatment. Preferred content:<br>-information about medication: 76%	NA

		-supporting messages: 70%<br>-how to reduce risk of relapse: 88%	
**Overcoming Addictions** [70]	Complex	Participants not willing to be randomized to online program only (without additional in person groups). Numbers of logins to website declined from 7.3 (average) the first three to 1.3 the next three months. Most often cited as helpful: social support and awareness reminders	Improvements in drinking outcomes were obtained in all test conditions, with no differences between conditions. Best effect for those who stopped drinking before entering the program. Internet fluency/ease no impact on results. 3 months results reported in Hester 2013
**A-CHESS** [71-77]	Complex	Higher rates of use than for colon cancer and asthma<br>Replaced 116 phones (among 170 participants in the experimental condition)<br>Changes in % using program from first week to fourth month:<br>-Any use: 94% to 78%<br>Modules related to<br>-competence 80% to 39%<br>-relatedness 91% to 76%<br>-autonomous motivation 84% to 66%<br>Use (averages) first 8 months:<br>-used 41% of days<br>-pressed "panic button" at least once: 72% (122 participants),<br>-of these, 98 participants then moved to at least one other page<br>-the "weekly check-in" module: 2.5 times a month<br>-4 modules entered per day<br>Used by adolescents (6 weeks):<br>-assessment module (EMA): 89% completed (not too long: 95%, easy to learn/use: 100%<br>-intervention module (EMI): accessed 78% of days (average), most used: recovery support, motivation, relaxation, social networking<br>Organizational prerequisites for implementation:<br>-strong leadership support<br>-passionate staff<br>-participant feedback reports and working groups engage participants<br>-issue in weekly meetings<br>-develop guidelines for use<br>-develop financial strategies for the sustained use of the intervention	Chih et al. (2014a): Combination of lapse history and current score on weekly recovery progress assessment predicted lapse risk in next 14 days. When risk of relapse was 5% or greater according to weekly score, model predicted relapse with 75% sensitivity and 88% specificity<br>Chih et al. (2014b): No difference in A-CHESS use between lapsers (N=51) and non-lapsers (N=91). Three profiles of A-CHESS users developed: inactive (33.9%), passive (49.3%), and active (14.8%). These groups did not differ in lapse rate (all p>.31).<br>Dennis et al. (2015): Adolescents who accessed 2+ EMI within one hour of EMA were less likely to use alcohol/drugs within the next 7 days, compared to those with less EMI use (OR=0.62).<br>Gustafson et al. (2014): Risky drinking days last 30 days: EXP=1.39 vs CTR=2.75 (p=0.003). Abstinence odds last 30 days at 8 and 12 months higher in EXP vs. CTR (p=.04). Difference predicted by number of pages viewed/ days in, but not by number of services used. Risky days results mediated by perceived competence, but not by relatedness or autonomous motivation.<br>Ford et al. (2015): NA<br>McTavish et al. (2012): NA<br><br>Other: Gustafson et al. (2014): No differences in negative consequences of drinking.<br><br>Glass et al. (2017): A-CHESS condition increased rate of outpatient SUD treatment during follow-up, but not mutual help attendance. The use of outpatient SUD treatment mediated effect of A-CHESS on risky drinking days but not abstinence.
**LBMI-A** (Location-Based Monitoring/ Intervention for Alcohol Use Disorders [78]	Complex	Launched all modules at least one time: 78%<br>Launched all tools at least one time: 68%<br>Use dropped rapidly first weeks<br>Website skills modules were not used.	Greater use of intervention associated with more severe drinking at baseline. Intervention produced larger increase in percent days abstinent than CTR in weeks 2-5 of the 6 week trial (large vs. moderate increases). Effects on percent heavy drinking days and drinks per week also favored intervention, but differences were smaller.
**Check-In Program** [79]	Complex	Used at least once: 92%<br>Required additional assistance beyond initial training: 35%<br>Returned first phone 44%<br>One replacement phone: 44%<br>During 12 weeks study (average):<br>-8.8 completions of functional analysis	mobile + education program (computer) produced higher mean weeks of abstinence at 3 months compared to standard methadone maintenance only (4.9 vs. 2.7; p=0.055)

		-21.1 completions of self-management<br>Mean scores on satisfaction<br>-interesting, useful, new information, generally satisfied: 75-80 (out of 100)<br>-easy to understand: 59<br>-clarified misunderstandings: 65<br>mobile + education program (computer) increased retention (84% vs 56%; p=0.031)	
**Health Call** [80, 81]	Complex	Used on 85% of days, not moderated by any tested variables<br>Very positive feedback on user interface and satisfaction with the content (80% or more)<br>Used on 95% of days (average of 3.1 minutes/day). Satisfaction with the program rated as 4.5 on a 1-5 scale	Number of Drinks per Day dropped from 9.3 to 3.9, with similar reduction in historical IVR control group. End of treatment abstinence rate was 25.6% in smartphone condition, vs. 16.3% in IVR comparison (NS)<br>MI+Health Call produced greater decrease in number days drug use and quantity of drugs at 2 months than MI only (IRR= 0.50, p< .01). No effects on alcohol use outcomes.
**MyFYR** (My First Year in Recovery) [82]	Complex	Of all 198 participants, 78% completed the program. Retention rate for those who relapsed was 70%.	Out of the 198 study participants, 51 had one or more positive urine test results. Of those, 21 (41.2%) tested positive only once, 19 (37.3%) had two positive urines, and 11 (21.6%) had three or more positive urines. Alcohol (41.0%) and opiates (28.0%) were the primary drugs that individuals relapsed on based on urinalysis results. Of the 107 individuals who relapsed at some point, 60 (56.1%) had no positive urine samples but self-reported one or more episodes of alcohol or drug use, or acknowledged use reported by a family member.
**Snow Control** [83]	Complex	281 registered online (via a website), 196 found to be eligible<br>Retention in program after:<br>-2 weeks: 44%<br>-6 weeks: 19%	No significant group x time interactions on severity of cocaine dependence, cocaine<br>craving, or depression. No effects on consumption of cocaine, cannabis, or alcohol (reduction of average weekly cocaine consumption, cocaine free days).
**Can Reduce** [84]	Complex	85% did intro module<br>Percentage using the intervention declined from 45% first week to 18% 6th week<br>Study follow up: 40%. 76% of those offered a chat room did not chat. Offering chat room did not increase use of modules	Days of cannabis significantly lower in chat vs. non chat condition (p= .02, d= .34), and in chat vs. wait list (p=.03, d= .20). No differences on quantity of cannabis, cannabis use disorder, and severity of dependence outcomes.<br>Other: No difference on mental health measures.
Tait [85]	Complex	Completed first module: 63%<br>Completed second module: 56%<br>Completed all three modules: 48%	Tait et al. (2014): 3 month follow-up (FU) data presented (FU rate only 50%).<br>Tait et al. (2015): 6 month FU rate about 50%. No group effects on SUD measures. "Help seeking" was higher in EXP than CTR (p=.02). Other: No difference on psychological distress. Significant difference in reduction in days of impairment ("days out of role") (p=0.001) and increased "help seeking" (p=0.02).
**Quit the Shit** [86]	Complex	NA	Greater reductions in use frequency and quantity in EXP vs. CTR (p< .001, d> .75).
**No intervention** [46]	Complex	Overall acceptance: 2.6 on a 1-5 scale, highest in adolescents (3.5), lower in SUD (2.3). Only 10% willing to pay for web-based continuing care,<br>Predictors of higher acceptance: younger age, more education, internet access, no prior ehealth experience, expected benefit, expected ease of use and attitude of significant others.	NA
